# Vision-Related Quality of Life in Patients With Atypical Optic Neuritis

**DOI:** 10.3389/fpain.2022.871269

**Published:** 2022-05-12

**Authors:** Zhaocai Jiang, Haiyan Qian, Shihui Wei

**Affiliations:** ^1^Beijing Jingmei Group General Hospital, Beijing, China; ^2^Senior Department of Ophthalmology, The Third Medical Center of People's Liberation Army (PLA) General Hospital, Beijing, China

**Keywords:** atypical optic neuritis, vision-related quality of life, NEI VFQ-39, demyelinating disease, pain

## Abstract

**Aim:**

To evaluate the vision-related quality of life (QOL) in patients with atypical optic neuritis through the 39-item National Eye Institute Visual Function Questionnaire (NEI VFQ-39).

**Methods:**

Fifty-seven patients with atypical optic neuritis were scheduled in the research unit from June 1 to December 31, 2019. Besides collecting the clinical data, NEI VFQ-39, the Chinese version, was applied to all subjects. The NEI VFQ-39 subscale item scores were compared among subgroups divided by different classifications, and a correlation analysis of the NEI VFQ-39 scores and best-corrected visual acuity (BCVA) for better-seeing and worse-seeing eyes was performed.

**Results:**

The mean age of scheduled patients was 34.3 ± 12.4 years, with the majority being female (70.2%). The mean composite score was 58.62 ± 17.62. Twenty-nine (50.9%) patients were affected binocularly, and the most subscale scores and composite scores of binocular incidences were lower than those of monocular incidence significantly. However, there was no statistically significant difference in composite scores between patients with and without periocular pain. The patients with worse BCVA in the better-seeing eye have lower scores than those with better BCVA in the better-seeing eye. Most NEI VFQ-39 scores have strong correlations with the BCVA for better-seeing and worse-seeing eyes.

**Conclusion:**

Atypical optic neuritis has a significant and comprehensive influence on patients' visual function outcomes and quality of life associated with vision. Improving their BCVA in the better-seeing eye can improve their vision-related QOL.

## Introduction

Optic neuritis (ON), an inflammatory demyelinating disease of one or both optic nerves, may occur as a single disease event, as a manifestation during multiple sclerosis (MS), or, maybe, as a result of the neuromyelitis optica (NMO). Typical ON is often characterized by acute or subacute loss of vision in the affected eye, a scotoma, and pain in the eyeball that often occurs with eye movement, but recovers vision within a few months spontaneously (1, 2). Though, patients with atypical ON have different clinical features with more serious or worse prognosis: bilateral loss of vision—simultaneous or sequential within 4 weeks; loss of vision to no perception of light with no early recovery; painless loss of vision to <6/60 with no early recovery; severe or persistent pain for >2 weeks since onset; visual loss progressing for >2weeks since the onset of visual symptoms; lack of any recovery >3 weeks after onset of visual symptoms; and worsening of vision after withdrawal of corticosteroids (3). Whether typical or atypical ON, many patients have lasting symptoms of visual impairment, which can affect their ability to perform various vision-specific functions in daily life.

Measurement of health-related QOL has been recognized as an important adjunct to clinical outcome measures. There are many QOL evaluation tools established. National Eye Institute's 39-Item Visual Function Questionnaire (NEI VFQ-39) is one of the tools, which is an instrument to assess self-reported visual impairment in studies of vision. It includes VFQ-25 and 14 optional items, which can greatly enhance the comparability of sub-scale scores across studies (4). The VFQ-25 has been used to track the outcome of many ocular diseases, including age-related macular degeneration, glaucoma, retinal vein occlusion, diabetic retinopathy, and ocular chemical burns (5–10). In 2000, Stephen et al. used NEI VFQ-51 to describe the QOL of patients several years after the onset of typical ON (11). However, until now, no studies have been performed to specifically assess the vision-related QOL in persons with atypical ON. Therefore, we assessed the QOL in patients with atypical optic neuritis by applying the NEI VFQ-39.

## Subjects and Methods

### Subjects

A total of 57 consecutive patients clinically diagnosed with atypical optic neuritis, based on atypical ON diagnostic criteria (3, 12), were scheduled for vision care at the Department of Neuro-Ophthalmology, Chinese PLA General Hospital, from June 1 to December 31, 2019. Patients enrolled into the study were similar in some aspects: they were 10 years of age and older, with no visual recovery over 3 weeks since onset, no other ocular diseases, and no diagnosis of a defined collagen vascular disease or neurological autoimmune disease at onset. The study was approved by the ethics committee of the hospital and conducted according to the tenets of the Declaration of Helsinki. Informed consent was obtained from all patients after the purpose of the study and the experimental procedures were carefully explained to them.

### Methods

#### Tests of Visual Function

All subjects received overall ophthalmologic examination, including best-corrected visual acuity (BCVA), slit-lamp biomicroscopy, direct ophthalmoscopy, fundus photography, and intraocular pressure (IOP, by Goldmann applanation tonometry). The visual evoked potential (VEP) was also obtained, when necessary. The BCVA was recorded 3 months since onset and was evaluated with the Snellen visual acuity chart. Approximations for visual acuity (VA) worse than 20/400 were as follows: counting fingers, 20/2,000; hand motions, 20/4,000; light perception, 20/8,000; and no light perception, 20/16,000 (6, 13).

#### Visual Function Questionnaire (VFQ-39)

All patients were requested to answer VFQ-39 3 months after onset and fill out the questionnaire by themselves. The research staff explained the questionnaire to the patients, verbally administered instruction, and assisted when requested. For the participants, whose eyesight was too poor to read, a single research staff member read the questionnaire for them, neutrally and uniformly, aided the patients' choices. The completed questionnaires were reviewed by the research staff.

The NEI VFQ-39 contains a reduced number of items within each subscale of the original 51-item NEI VFQ (4, 14). Each item is assigned to one of the 12 subscales: general health, general vision, ocular pain, near activities, distance activities, social functioning, mental health, role difficulties, dependency, driving, color vision, and peripheral vision. Answers to each question on the VFQ-39 were transformed to a 100-point scale, in which 100 stands for the best possible score or the minimal subjective impairment, and 0 represents the worst or the maximal subjective impairment. The composite VFQ-39 score is the mean score of all items except for the general health item.

The VFQ-39 used in this study was a Chinese version, with modifications to suit the Chinese culture and way of life. The modified NEI VFQ-39 questionnaire has been assessed for reliability and validity, and it has been proven to accurately measure VR-QOL in Chinese individuals (15, 16).

#### Statistical Analysis

All statistical analyses were performed with the SPSS version 19.0 software platform (SPSS Inc., Chicago, IL). Descriptive statistics were obtained with the application of frequency, percentage, mean, and standard deviations (SDs). We counted the mean scores and SDs for each VFQ-39 subscale and composite score and applied the Mann–Whitney *U*-test to compare each subscale and composite score between patients with monocular incidence and those with binocular incidence, and between patients with Periocular pain and those without Periocular pain. Further analysis on the mean VFQ-39 scores by BCVA in the better-seeing eye were made through non-parametric ANOVA Kruskal–Wallis H (K). Non-parametric correlation analysis of the NEI VFQ-39 scores and BCVA for better-seeing and worse-seeing eyes was performed using the Spearman test. All tests were considered statistically significant at *P* < 0.05.

## Results

A total of 57 patients (86 eyes) participated in the study, and 29 patients were suffering from bilateral affection. The characteristics of these subjects are shown in Table 1: age (10–63 y; mean, 34.3 ± 12.4 y); sex (male/female, 17/40); periocular pain (26, 45.6%), the mean age of patients with and without periocular pain was 28.5 ± 12.2 y and 39.2 ± 10.5 y, respectively (*P* = 0.001); VEP [Pattern VEP (PVEP) 32, 56.1%, 45 eyes; Flash VEP (FVEP) 18, 31.6%, 32 eyes]. The mean latency of P100 for PVEP was 131.05 ± 19.15 ms, which was delayed, as compared to normal values of PVEP in China (96.67 ± 4.10, *P* < 0.001) (17). The mean latency of P2 for FVEP was 131.77 ± 27.50 ms, which was also delayed, as compared to normal values of FVEP in China (78.29 ± 9.85, *P* < 0.001) (17). However, there were no correlations between the latency and NEI VFQ-39 scores.

**Table 1:** Clinical characteristics of all enrolled subjects.

**Clinical characteristics (*n* = 57)**	
Sex	
Male	17 (29.8%)
Female	40 (70.2%)
Age (Mean ± SD) (y)	34.3 ± 12.4
Periocular pain	
Have	26 (45.6%)
None	31 (54.4%)
VEP	
None	7 (12.3%), 9 eyes
PVEP	32 (56.1%), 45 eyes
FVEP	18 (31.6%), 32 eyes

The mean scores of each VFQ-39 subscale are all listed in Table 2. The mean composite score was 58.62 ± 17.62. We eliminated the driving subscale because of the high missing rate (37/57, 64.9%) when we counted the composite score and did further analysis according to the previous research (15). The score for mental health (46.25 ± 21.44) was the lowest, followed by the scores for general vision (47.41 ± 20.47), role difficulties (48.81 ± 23.18), and dependency (49.79 ± 26.51).

**Table 2:** Mean scores for each subscale and composite scores of VFQ-39 in all subjects.

**Subscale**	***n***	**Mean**	**SD**	**Median**	**Range**
General health	57	59.95	19.21	65	15–95
General vision	57	47.41	20.47	45	0–100
Ocular pain	57	66.89	20.39	62.5	12.5–100
Near activity	57	60.74	24.01	66.7	12.5–100
Distance activity	57	59.67	24.64	66.7	6.25–100
Social function	57	71.56	24.6	75	16.7–100
Mental health	57	46.25	21.44	45	0–95
Role difficulties	57	48.81	23.18	50	0–93.75
Dependency	57	49.79	26.51	50	0–100
Driving	20	17.08	34.35	0	0–100
Color vision	57	72.36	29.38	75	0–100
Peripheral vision	57	61.4	25.47	75	0–100
Composite score	57	58.62	17.64	58.25	20–91.4

The analyses of each subscale score and composite score between patients with monocular incidence and those with binocular incidence, and between patients with periocular pain and those without periocular pain are shown in Tables 3, 4. In addition to the scores for general health, ocular pain, and mental health, all scores in patients with binocular incidence were lower significantly. Nonetheless, there was no statistical significance between patients with periocular pain and those without periocular pain. Table 5 shows that the difference in subscale scores and composite scores, except those for general health and ocular pain subscales, had statistical significance between group 1 and group 2, and group 3 divided by BCVA in the better-seeing eye. However, there was no statistical significance between group 2 and group 3, except for the scores for peripheral vision.

**Table 3:** Analysis of each subscale and composite score between patients with monocular incidence and binocular incidence.

**Subscale**	**Binocular injury (*n* = 29)**	**Monocular injury (*n* = 28)**	***P***
	**Mean ±SD**	**Mean ±SD**	
General health	58.02 ± 21.43	61.95 ± 16.79	0.564
General vision	41.90 ± 23.81	53.13 ± 14.67	0.021^a^
Ocular pain	63.79 ± 18.10	70.09 ± 22.40	0.181
Near activity	47.10 ± 21.65	74.86 ± 17.40	0.000^b^
Distance activity	48.66 ± 24.26	71.08 ± 19.59	0.001^a^
Social function	62.21 ± 26.34	81.24 ± 18.55	0.004^a^
Mental health	43.84 ± 21.90	48.75 ± 21.07	0.165
Role difficulties	38.60 ± 22.40	59.37 ± 19.13	0.001^a^
Dependency	42.25 ± 25.91	57.59 ± 25.25	0.016^a^
Color vision	63.79 ± 31.75	81.25 ± 24.18	0.031^a^
Peripheral vision	52.59 ± 27.82	70.54 ± 19.31	0.008^a^
Composite score	51.15 ± 17.48	66.35 ± 14.37	0.000^b^

a *P <0.05*;

b *P <0.001*.

**Table 4:** Analysis of each subscale and composite score between patients with and without periocular pain.

**Subscale**	**Periocular pain (*n* = 26)**	**No periocular pain (*n* = 31)**	***P***
	**Mean ±SD**	**Mean ±SD**	
General health	58.56 ± 17.42	61.12 ± 20.82	0.612
General vision	50.86 ± 24.77	44.52 ± 15.88	0.249
Ocular pain	67.31 ± 22.66	66.53 ± 18.65	0.702
Near activity	61.38 ± 25.18	60.21 ± 23.39	0.712
Distance activity	58.04 ± 25.52	61.05 ± 24.21	0.653
Social function	70.99 ± 24.95	72.04 ± 24.70	0.884
Mental health	47.50 ± 26.84	45.20 ± 15.98	0.828
Role difficulties	47.60 ± 26.34	49.82 ± 20.55	0.936
Dependency	52.16 ± 33.72	47.79 ± 18.85	0.735
Color vision	73.08 ± 29.94	71.77 ± 29.40	0.833
Peripheral vision	63.46 ± 26.67	59.68 ± 24.73	0.51
Composite score	59.18 ± 20.63	58.16 ± 15.01	0.773

^a^*P <0.05*;

^b^*P <0.001*.

**Table 5:** Analysis of composite score and subscale scores among groups divided by best-corrected visual acuity (BCVA) in the better-seeing eye.

**Subscale**	**Group 1 (*n* = 9)**	**Group 2 (*n* = 12)**	**Group 3 (*n* = 36)**	***P***		
	**Mean ±SD**	**Mean ±SD**	**Mean ±SD**	**A**	**B**	**C**
General health	50.56 ± 18.40	56.67 ± 22.72	63.39 ± 17.68	0.467	0.074	0.291
General vision	21.67 ± 14.79	48.33 ± 22.29	53.54 ± 15.97	0.007^a^	0.000^b^	0.376
Ocular pain	70.83 ± 18.75	62.50 ± 19.21	67.36 ± 21.40	0.363	0.653	0.482
Near activity	26.39 ± 08.08	57.60 ± 20.65	70.37 ± 19.26	0.010^a^	0.000^b^	0.113
Distance activity	25.69 ± 10.81	55.63 ± 23.81	69.52 ± 19.08	0.011^a^	0.000^b^	0.134
Social function	38.91 ± 15.64	72.91 ± 26.38	79.27 ± 18.91	0.005^a^	0.000^b^	0.45
Mental health	30.00 ± 09.01	49.17 ± 22.65	49.34 ± 21.80	0.029^a^	0.005^a^	0.816
Role difficulties	20.87 ± 08.89	47.43 ± 20.70	56.25 ± 21.18	0.011^a^	0.000^b^	0.228
Dependency	20.17 ± 09.80	49.48 ± 27.75	57.29 ± 24.02	0.006^a^	0.000^b^	0.335
Color vision	47.22 ± 31.73	75.00 ± 31.98	77.78 ± 25.20	0.034^a^	0.010^a^	0.922
Peripheral vision	38.89 ± 22.05	52.08 ± 29.11	70.14 ± 20.55	0.238	0.001^a^	0.039^a^
Composite score	35.57 ± 08.06	56.98 ± 17.21	64.93 ± 14.53	0.009^a^	0.000^b^	0.174

*Group 1: BCVA of better-seeing eye <20/200; Group 2: 20/200 < = BCVA of better-seeing eye < = 20/32; Group 3: BCVA of better-seeing eye > 20/32. A: group 1 vs. group 2; B: group 1 vs. group 3; C: group 2 vs. group 3*.

a *P <0.05*;

b *P <0.001*.

Table 6 shows that most NEI VFQ-39 scores have strong correlations with the BCVA for better-seeing and worse-seeing eyes. However, the correlations between VFQ-39 scores and BCVA in the better-seeing eyes were stronger than in the worse-seeing eyes. All VFQ-39 scores have significant correlations with BCVA in better-seeing eyes, except for general health and ocular pain, similar to worse-seeing eyes except of general health, ocular pain, and peripheral vision.

**Table 6:** Correlations analysis of the NEI VFQ-39 scores and BCVA for better-seeing and worse-seeing eyes.

**VFQ-39 scores**	**BCVA (better-seeing eye)**		**BCVA (worse-seeing eye)**	
	**CC**	***P***	**CC**	***P***
General health	0.132	0.328	0.144	0.285
General vision	0.54	0.000^b^	0.409	0.002^a^
Ocular pain	0.151	0.263	−0.043	0.751
Near activity	0.665	0.000^b^	0.282	0.034^a^
Distance activity	0.637	0.000^b^	0.288	0.030^a^
Social function	0.541	0.000^b^	0.429	0.001^a^
Mental health	0.394	0.002^a^	0.389	0.003^a^
Role difficulties	0.639	0.000^b^	0.281	0.034^a^
Dependency	0.551	0.000^b^	0.406	0.002^a^
Color vision	0.367	0.005^a^	0.344	0.009^a^
Peripheral vision	0.518	0.000^b^	0.18	0.179
Composite score	0.631	0.000^b^	0.389	0.003^a^

*CC, Correlation Coefficient*;

a *P <0.05*;

b *P <0.001*.

## Discussion

Atypical optic neuritis refers to a group of inflammatory optic neuropathies, which requires corticosteroids to induce recovery and often continued immunosuppression to maintain remission (3). The disease not only caused visual damage but also influenced their ability to perform various activities in daily life. Therefore, it is very necessary to investigate the vision-related QOL in patients with atypical optic neuritis by the reliable and effective tool: NEI VFQ-39 (15).

The VEP is used primarily to measure the functional integrity of the visual pathways from the retina *via* the optic nerves to the visual cortex of the brain (18). It is an important objective diagnostic tool in demyelinating diseases, such as ON. In our study, the latencies of patients' VEP, both PVEP and FVEP, were delayed, as compared to normal values in China. This supported the diagnosis of ON. The latency of P100 and P2, however, did not reflect the scores for NEI VFQ-39.

The NEI VFQ-39 composite scores in patients with atypical optic neuritis were lower, compared with age-related macular degeneration (9), dry eye syndrome (19), and glaucoma (20). In addition, the scores for mental health, general vision, role difficulties, and dependency were the lowest. These results demonstrated that atypical optic neuritis not only caused visual acuity impact, but also impaired the patients' vision-related QOL and psychological health. More substantial assistance should be provided for these patients not only for their physical health, but also for their QOL and mental health.

This study showed that the scores of each VFQ-39 subscale and composite were significantly lower in patients with binocular incidence than those with monocular incidence, except for the scores of general health, ocular pain, and mental health. These results are consistent with previous studies (10, 21). In our study, the scores for near activity, distance activity, and role difficulties were statistically different, which meant that patients with bilateral incidence have more difficulty in performing vision-related daily activities and of lower vision-related QOL than those with unilateral incidence. However, there was no statistical significance between patients with periocular pain and those without periocular pain. The reasons may be that the periocular pain was very slight, and the patients did not pay much attention to it as long as they could see things clearly; or the pain threshold of some patients was very high, which did not show much influence on their daily-life activities. From detail analysis, we found that older patients could not feel eye pain clearly due to their degradation of organs, which led to a slow response to pain; although younger patients could sense pain, it had no significant effects to daily life, probably because of their higher tolerance to it.

The patients with BCVA of better-seeing eye with <20/200 have lower scores than those with 20/200 ≤ BCVA of better-seeing eye with ≤ 20/32 and BCVA of better-seeing eye with >20/32. When their BCVA of better-seeing eye was better than 20/200, the scores were not statistically different. That means, in other words, severe visual impairment meant poor vision-related QOL. This result indicates that appropriate treatment for atypical optic neuritis to prevent vision loss is critical for patients to maintain their QOL, and patients with a severe impaired vision need more support.

This research indicated that BCVA for both the better-seeing eyes and the worse-seeing eyes significantly correlated with most of the VFQ-39 scores. Also, the correlations between VFQ-39 scores and BCVA in the better-seeing eyes were stronger than the worse-seeing eyes. That means the visual functions not only in the better-seeing eyes, but the worse-seeing eyes are also important to patients' QOL.

There were some limitations in our study. First, the sample size of this study was small; Second, all the patients were only from one hospital, and there might exist selection bias; Third, the time span of this study was only 6 months; Moreover, we did not assess the VFQ-39 scores before and after treatment, because many patients had treatment in other hospital before they came to our hospital.

In conclusion, patients with atypical ON have more severe symptoms and poorer prognosis than patients with typical ON (3). Atypical ON can affect daily life associated with vision, which leads to descent of QOL. According to the results of the present and previous study (14, 22), the VFQ-39 has been regarded as an effective tool to assess vision function and vision-related QOL. We can see from our study that patients with BCVA of more than 20/200 had fewer influence on their QOL, so ophthalmologists should try their best to improve patients' BCVA. To overcome the shortcoming of our study, a larger scale, multicenter, random, and controlled clinical research is expected.

## Data Availability Statement

The original contributions presented in the study are included in the article/supplementary material, further inquiries can be directed to the corresponding author/s.

## Ethics Statement

The study was reviewed and approved by the Senior Department of Ophthalmology, the Third Medical Center of PLA General Hospital and conducted according to the tenets of the Declaration of Helsinki. Written informed consent was obtained from all patients after the purpose of the study and the experimental procedures were carefully explained to them.

## Author Contributions

ZJ is responsible for the writing and the statistical analysis of the article. SW is the reviewer of the article. HQ is responsible for the communication with patients. All authors contributed to the article and approved the submitted version.

## Conflict of Interest

The authors declare that the research was conducted in the absence of any commercial or financial relationships that could be construed as a potential conflict of interest.

## Publisher's Note

All claims expressed in this article are solely those of the authors and do not necessarily represent those of their affiliated organizations, or those of the publisher, the editors and the reviewers. Any product that may be evaluated in this article, or claim that may be made by its manufacturer, is not guaranteed or endorsed by the publisher.
